# Supplementary material for: Chromothripsis during telomere crisis is independent of NHEJ, and consistent with a replicative origin
Source: Genome Res. 2019 May;29(5):737–49. doi: 10.1101/gr.240705.118 (PMC6499312; doi:10.1101/gr.240705.118)
Supplement: Supplemental Material [file supp_gr.240705.118_Supplemental_file_1.zip › contigs/annotated_contigs/DB116/contig.3.DB116_length_540_mean_cov_8.61111111111.docx]

**DB116_length_540_mean_cov_8.61111111111**

```
CCCCTTTACAAAATGTAAGAACAAGAAATGTAACAATAAAACAAATACAGTGGTGGGGAAGGAACAAGAGCAGGGGTCAGCAGTGTAAT
 >chr13:39960384-39960591 + E=4e-108
TCTTCAGACCCTGGAAACTCAAGGAGTACATTCTTTAGTCTACAAAAAAAAGGCTTGGTTGCATTCTAGTAAACCACACCTCCTATCAA

AGTGCTTTCTGGAAACTGAGGCACAG|AG|CCAGATTTCCAGTCACCATTTGTCTAAGAATGACACTGCTTTAGCCACAGGGAAGTCTC
                           >chr13:39961967-39962058 + E=4e-43
CCACTGCTGCCTGTGACTAACGACAAGGGA|ACG|GCAACACCTTTCTAACCCCAGTAGTTAAGGGCATCTTAATTCTACCATTTCACT
                                  >chr13:39962644-39962886 + E=5e-134
TCTTTAGGACTCAGTTTTCCTTATCTGTAAAATGGGGGAAAATTATGAGGAGGGGGAGGTAACACCTTGAACATTTATTTTTATTTCTG

TAACAGTGACTTTATGAAAACAAAGTAGACCATCATTACATTAGGATGCACAGTGTTGTGGGAGAGATTTGCAAAACTTCCACTGGAAT

TGCAGGGCAG
```
